# The Park Prescription Study: Development of a community-based physical activity intervention for a multi-ethnic Asian population

**DOI:** 10.1371/journal.pone.0218247

**Published:** 2019-06-11

**Authors:** Léonie Uijtdewilligen, Clarice Nhat-Hien Waters, Su Aw, Mee Lian Wong, Angelia Sia, Anbumalar Ramiah, Michael Wong, Falk Müller-Riemenschneider

**Affiliations:** 1 Saw Swee Hock School of Public Health, National University of Singapore, Singapore; 2 Centre for Urban Greenery & Ecology, National Parks Board Singapore, Singapore; 3 Health for Life Centre, Alexandra Health Pte Ltd, Singapore; 4 Institute for Social Medicine, Epidemiology and Health Economics, Charite Univeristy Medical Centre, Berlin, Germany; Emory University, UNITED STATES

## Abstract

This mixed-methods study aims to inform the development of a ‘Park Prescription’ intervention, including face-to-face counseling on physical activity and park use and providing weekly structured exercise sessions in the park to promote physical activity. Participants aged 40–65 years were recruited from regional health screening events in Singapore where they completed a questionnaire (N = 97) and consented to focus group (FG) participation (N = 16). The questionnaire assessed current park use, and the type, duration, and intensity of park-based activities that would be of interest. FGs explored the barriers and facilitators of physical activity (in parks). Short interviews (N = 16) with ‘doers’, i.e., people already engaging in park-based physical activity, identified motivational factors and ways to overcome common barriers. Participants acknowledged the health benefits of parks and valued them because of their pleasant landscapes, greenery and facilities. However, few participants engaged in physical activity at the parks, because they were too busy or too tired. Participants mostly indicated doing informal activities, such as walking, cycling or playing traditional Asian games when using the parks for exercise. A variety of low-to-moderate intensity park-based activities such as walking, cycling or aerobics were of interest to participants who expressed the willingness to engage in structured exercise sessions on weekday evenings or weekend mornings. Strategies to increase physical activity in parks included: encourage planning, create social support, identify alternatives for bad weather, improve proximity/accessibility to parks and park safety. The effectiveness of the Park Prescription intervention in promoting physical activity, park use, as well as physical and mental well-being will be tested in a one-year Randomized Controlled Trial.

## Introduction

Being physically inactive, defined as accumulating less than 150 minutes of moderate- to vigorous-intensity physical activity per week, is a significant risk factor for developing non-communicable, chronic diseases such as stroke, diabetes, and cancer [1–3]. Physical inactivity is also one of the 10 leading risk factors for global mortality [4]. The World Health Organization (WHO) estimated the worldwide physical inactivity prevalence among adults to be 23.3%, with varying percentages across WHO regions; from 32.4% in the Americas to 14.7% in South-East Asia [5]. But also within these regions prevalence rates of physical inactivity differ. For example, research among a Singapore sample showed that over 26% of adults were not sufficiently physically active and only 24% engaged in regular leisure-time physical activity [6]. To develop evidence-based interventions, countries have been monitoring their populations’ physical activity levels more closely and research on the correlates of physical activity has increased, also among low- and middle-income countries [7] where the health burden of non-communicable diseases is disproportionately high compared to high-income countries [8]. There is a high demand for novel and effective programs to mitigate the global pandemic of physical inactivity.

Healthcare systems may be good platforms to implement and roll out strategies that increase physical activity levels for chronic disease prevention [9]. One of the objectives of the Healthy People 2020, a 10-year agenda of the United States (US) Department of Health and Human Services for improving public health, is to increase the proportion of physician visits that include physical activity counseling or education [10]. Similarly, WHO has prioritized ‘the promotion of physical activity for all adults from all social groups as part of daily life, […] through the healthcare system’ in their recently released physical activity strategy document [11]. The *Exercise is Medicine* (EIM) initiative is a well-known healthcare-based strategy for promoting physical activity. The American College of Sports Medicine (ACSM) developed EIM as an alternative form of treatment or preventive medicine compared to traditional medicine-based prescriptions where healthcare providers refer their patients to EIM certified exercise programs [12]. Healthcare providers are encouraged to use the EIM physical activity prescription pad, which is a basic exercise prescription in an easy-to-use and printable format providing space for written recommendations on the type, frequency and duration of physical activity a patient should engage in.

Along the lines of EIM, the US Centers for Disease Control and Prevention and the US National Recreation and Park Association collaborated to develop the ‘National Park Prescriptions Initiative’ that brings together healthcare providers and stakeholders of park associations in order to improve physical and mental health among individuals and communities [13]. A definition of ‘Park Prescriptions’ was formalized in 2013 during the National Park Prescriptions Initiative Convening, encompassing ‘‘programes that are designed by healthcare providers and relevant community partners to utilize parks, trails, and open space to improve individual and community health”. A ‘Park Prescription’ could, compared to a normal EIM prescription, incorporate the park-context in recommendations on physical activity type by highlighting suitable walking trails or available exercise corners in parks. Practitioners may further point out nearby parks where people can be active and provide park maps on which physical activity opportunities are indicated. Throughout the prescription process practitioners could emphasize the additional health benefits of the natural environments that parks are.

Natural environments/(urban) green spaces have been associated with several beneficial health effects, including reduced cardiovascular mortality [14,15], lower Type 2 diabetes risk [16] and improved mental health and well-being [14,17]. The mechanisms thought to underlie these associations include: parks provide a setting for community engagement, greenery provides restorative sensory effect, spiritual values enhance from being in direct contact with nature and physical activity and leisure recreation increases in residents when parks and green spaces are present in the neighborhood [18]. Evidence suggests that visiting local green spaces increases the odds to achieve the recommended amount of physical activity [19] and that neighborhood green protects against physical activity decline in older adults [20]. Presence of and access to green spaces has further been linked to more leisure time physical activity engagement in suburban residential areas and more active commuting in urban residential areas [21]. Several studies report promising outcomes for interventions that combine physical activity with green space exposure. For example, a systematic review by Hunter and colleagues showed that physical activity programs in combination with physical changes to the built environment (e.g., improvements of walking paths, gyms and landscaping) are likely to positively influence physical activity levels. However, the authors also noted that robust evaluations of such programs are required [22]. Han and colleagues [23] examined the impact of providing free exercise classes in low-income neighborhood parks and found that the classes increased participants’ moderate-to-vigorous physical activity. Another study by Marselle et al. [24] reported improved well-being, including lower depression rates and lower perceived stress in groups of people who attended nature walks compared to those who did not. According to another study by Calogiuri et al. [25] outdoor exercises versus indoor exercises increased positive feelings in office employees.

To the best of our knowledge, however, there is a lack of methodologically rigorous research studies that have looked specifically into combining exercise/physical activity prescription with a focus on the use of parks and green spaces. Most studies in the field of physical activity and green spaces did not integrate a healthcare system, did not use the ‘act of prescribing’ to improve participants’ behavior and health, or were conducted in Western contexts only.

This mixed-methods study, which is named the Park Prescription Study, aims to inform the development of a ‘Park Prescription’ intervention, including face-to-face counseling on physical activity and park use and providing weekly structured exercise sessions in the park to promote physical activity among inactive individuals from a northern community of Singapore. It was designed to better understand park use and physical activity behaviors in an Asian setting, making it an original contribution to the research field. For the purpose of this study, a collaborating team was formed with researchers and staff of the [details omitted for double-blind reviewing].

## Materials and methods

To understand participants’ park use, their physical activity behavior (in general and with the focus on park-based physical activities) and common barriers and facilitators to physical activity engagement and visiting parks, this mixed-method sequential explanatory study [26] includes three inter-related components:

- *Component 1*: An anonymous self-administered questionnaire;
- *Component 2*: A focus group (FG);
- *Component 3*: A series of short interviews with people who already engaged in physical activity in the park. This ‘doers’ research [27] provided input for enhancing non-doers motivations and helping them overcome their barriers with practical tips shared by their peers.

The qualitative data from component 2 (FGs) and component 3 (interviews) were used to further explain and interpret the quantitative findings from component 1 (questionnaire).

Details on the recruitment efforts and procedures for all components are described below. This research has been approved by the National Healthcare Group Domain Specific Review Board (DSRB) in Singapore [2015/0015-Park Prescription Study] and has been conducted in full accordance with the 1964 Helsinki declaration and its later amendments.

### Recruitment component 1: Quantitative questionnaire

Participants aged 40–65 years were recruited from health screening events organized by one large regional hospital located in the North of Singapore. These screenings targeted Singaporean nationals and permanent residents living in the North of Singapore, with the objective of early detection of diseases, increasing health awareness and encouraging positive healthy lifestyle changes. The hospital collaborated with community partners, such as religious institutions, to recruit residents for the screening events. The events were advertised on websites, through posters, and mailed flyers. All events followed a systematic approach through a series of health screenings at common community outdoor open spaces or community centres, followed by separate sessions during which residents picked-up their health reports [28]. On the screening day, residents had their measurements taken which included fasting blood tests for blood cholesterol and glucose, measurements of blood pressure, height and weight and a questionnaire to assess their current health status and health behaviors (e.g., smoking, physical activity). Approximately one week later, residents’ picked-up their health reports and based on their screening results were directed to a health seminar provided by the Singapore Health Promotion Board to assist them in interpreting their screening results as well as to give them relevant health promotion education. A nominal fee of $2 was charged to each individual as co-payment for the screening.

We recruited residents from seven screening events between May and August 2015. For the initial four screenings all eligibility criteria listed below applied. For the remaining three screenings conducted from end of July to end of August, we only applied selection criteria one to four, due to logistical constraints.

Eligibility criteria:

1. Provide informed consent;
2. 40–65 years old;
3. Singaporean national or permanent resident;
4. Report exercising <30 minutes per week;
5. Pass the Physical Activity Readiness Questionnaire (PAR-Q) [29];
6. Blood pressure of ≤139 mmHG (systolic) over ≤89 mmHG (diastolic);
7. Fasting glucose levels of ≤6.0 mmol/L.

During both the screening and report collection days, eligible residents were directed to the Park Prescription booth manned by trained [details omitted for double-blind reviewing] researchers. They were informed on the study content and received the participant information and consent form at the screening sessions. Eligible residents subsequently provided written informed consent at the report collection day if they chose to participate in the study.

Data collection through completion of the questionnaire took place on the report collection day for all participants. Questionnaires were provided in English, Malay and Chinese (S1–S3 Supporting information). Participants were given the questionnaire in the language they were most comfortable with. They received vouchers worth S$25 for completing the questionnaire. The target was to recruit a convenience sample of 50–100 participants.

### Recruitment component 2: Focus groups

Participants for the FGs were recruited from the same screening sessions described under component 1, and the same selection criteria applied. Information on the FGs was included in the original participant information and consent form. Although the option of only attending a FG was available, all FG participants had completed the questionnaire prior to their FG participation. Due to scheduling logistics, the FG was not held on the report collection day but on a subsequent weekend between August and September 2015 that was most convenient to participants. Participants who were interested in attending a FG were contacted shortly after the report collection day and informed about the specific date/time/venue of the FG. Verbal consent from each participant was obtained prior to the start of the FG. Vouchers worth S$25 were given for participation in a FG. The target was to conduct between two to four FGs in total, with a maximum of eight participants per group.

### Recruitment component 3: Doers research

Participants for the short individual interviews were recruited between December 2015 and January 2016, while being physically active at parks located in the North of Singapore. They were not part of the questionnaire or FGs sample. Doers were selected based on a matrix of their age and gender profiles: 40-<55-year-old males, 40-<55-year-old females, 55-<65-year-old males and 55-<65-year-old females. They were randomly approached by a trained interviewer from [details omitted for double-blind reviewing] while they were either participating in a structured-exercise session in the park on Sunday mornings or were performing physical activity at the park on their own (e.g., walking at a brisk pace, running, cycling, playing active games, etc.) on weekday mornings or afternoons. The interviewer explained the study content, provided an information letter and asked whether they were interested to participate. All participants provided verbal consent. The interviews took place while standing or walking along with the participants. This mobile interviewing technique, compared to a seated face-to-face situation, puts participant at ease and may add more insightful details to the conversation. While sharing their views, participants can directly reflect on their surroundings (i.e., the park) and their relationship to it. It is also a time- and resource efficient way of collecting information [30]. Participants received a simple hand towel and cap as appreciation for their time. The target was to recruit 16 participants, with four participants in each age and gender profile.

### Measures

#### Component 1: Quantitative questionnaire

Socio-demographic information included age, gender, ethnicity, marital status, level of education, and employment status. The questions on park use included the number of times participants visited any park in their local area over the past month, the physical and social activities they had engaged in during their last park visit and common reasons for not visiting parks. These questions were modified from a park survey developed by Leslie et al [31,32]. For eight different park-based physical activities, participants indicated their interest on a 4-point scale ranging from ‘not interested at all’ to ‘very interested’. They could also state other physical activities they would enjoy doing in parks in an open-ended format. Additional questions included how often they would visit parks to engage in such physical activities, how long they would do the activities for, and at what intensity they would enjoy doing them. These questions were developed specifically for the current research study to be able to inform he structured-exercise component of the Park Prescription intervention.

#### Component 2: Focus groups topic guide

The topic guide for the FGs was grounded in the PRECEDE framework [33]. As part of the sequential explanatory mixed-method design, the topic guide was refined based on preliminary findings from the quantitative questionnaire results. It included questions on motivating factors and perceived benefits, reinforcing factors, enabling factors and barriers for physical activity and physical activity in parks that were organized by the following topics and sub-domains:

- Topic 1: Participant’s physical activity
  ○ Domain 1: Perceived current physical activity and health
  ○ Domain 2: Intention and motivations to increase physical activity
  ○ Domain 3: Barriers to increasing physical activity
- Topic 2: Parks for physical activity
  ○ Domain 1: Neighborhood parks
  ○ Domain 2: Physical activity programs within parks
  ○ Domain 3: Barriers to physical activity within parks

With these topics and domains, we aimed to identify parks in the community that would be suitable for physical activity. We further aimed to identify any perceived facilitators and barriers for engaging in physical activity in the parks, suggestions for park improvement to motivate more physical activities, as well as get input on how to best structure an exercise program within a designated local park for individuals within their neighborhood. The full topic guide is available as S4 Supporting information.

Prior to the FG, the moderator informed participants on the aim, its interactive nature, and the definition of key concepts so participants could understand the topics for discussion. Anonymous socio-demographic data (age, gender, and ethnicity) were collected. Moderators did not have a prior relationship with participants. All moderators conducted or at least attended several FGs before leading a FG for the current study. They were a female postdoc researcher fluent in English, a female PhD student and a male research assistant fluent in both English and Chinese. Each of them took up the moderator role in turn. FGs were conducted in both English and Chinese. They were audio taped and a second staff researcher was present to take notes. The FGs were held in a closed meeting room at [details omitted for double-blind reviewing] with only [details omitted for double-blind reviewing] staff and participants present or at one of the health screening venues where hospital staff and non-participants of the study were present as well.

#### Component 3: Doers interviews

The doers interviews, comprising five open-ended questions (S5 Supporting information), aimed to explore the benefits/reinforcing factors, enabling factors/self-efficacy, barriers and strategies for overcoming specific barriers (i.e., lack of time, feeling too tired, weather concerns) related to being physically active in the park [27]. The questions on specific barriers were designed based on issues that were consistently mentioned in the FGs. The scope of the questions was kept to a minimum to reduce the disruption of participant’s physical activity routine. The interviewers were two male research assistants who recorded the key points of their discussion with participants in writing only. No follow-up interviews were carried out and the interviewers did not have a prior relationship with participants. Participants were asked to verify their age, gender and ethnicity prior to the interview.

### Outcomes and analysis

#### Component 1: Quantitative questionnaire

Participants’ responses on the number of times they visited any park in their local area over the past month were collapsed into the following categories: ‘never’, ‘once or twice’, ‘three to seven times’ and ‘eight times or more’. Categories for activities that participants had engaged in while being in the park were collapsed for ‘active sport’ (e.g., cricket, football, etc.) and ‘informal activities’ (cycling, martial arts, etc.). For the items on preferred frequency (i.e., ‘once a week’, ‘twice a week’, ‘three times a week or more’) and intensity of park-based activities (i.e., ‘light intensity only’, ‘up to moderate intensity’, ‘up to vigorous intensity’) the categories were kept similar to the answering options in the original questionnaire. Answering options for the preferred duration of park-based activities were collapsed into ‘15–30 minutes’, ‘45 minutes’ and ‘60 minutes or more’. Participant responses on their interest in eight different park-based activities were dichotomized as ‘not interested’ and ‘interested’. For the item on common reasons for not visiting parks, the categories were kept similar to the answering options in the original questionnaire. Written statements from open-ended formats were checked and re-classified within the fixed questionnaire categories if appropriate. For items considered not re-classifiable, new categories were derived. Descriptive statistics (means and SD, or proportions) were calculated for all variables and analyses were performed in IBM SPSS Statistics version 24.

#### Component 2: Focus groups

All FGs were transcribed verbatim in English by a bilingual research assistant. The FGs conducted in Chinese were translated into English and back-translated into Chinese to ensure consistency in meaning. Transcripts were checked for transcription errors by another bilingual research assistant. The transcripts were not returned to participants for commenting. Thematic analysis was conducted by two independent reviewers using *a priori* codes previously established on the PRECEDE model [33,34]. For text that could not be coded with pre-established codes, reviewers added new codes to the coding scheme, using an inductive data-driven approach [35]. To improve inter-coder reliability, pre-established codes were clearly organized in a codebook and reviewers were instructed to keep the number of new codes to a minimum [36]. Reviewers independently read and coded the transcripts before coming together to categorize the codes into themes. They met a total of three times to discuss codes and themes and reach consensus. They independently re-coded the data after these meetings if necessary [36]. The FGs were used to provide more details and in-depth explanations to the quantitative questionnaire findings [26]. Themes were matched to quantitative questionnaire findings for reinforcement purposes and to further inform intervention development.

#### Component 3: Doers research

The interviewer systematically summarized the notes taken during and after the doers interviews by participant and by question. These summaries were not returned to participants for commenting. To reduce reflexivity bias [37] three reviewers of different gender, age group and cultural background independently read and categorized participant responses and views. The topic of the five interview questions (i.e., benefits/reinforcing factors, enabling factors/self-efficacy, barriers and overcoming specific barriers) served as a priori themes for categorizing the data. The three reviewers met a total of two times to discuss their data categorization per theme and reach consensus.

No special software was used to analyze the qualitative FG or doers interview data. Participants were not asked to provide feedback on the study findings.

## Results

**Fig 1:**
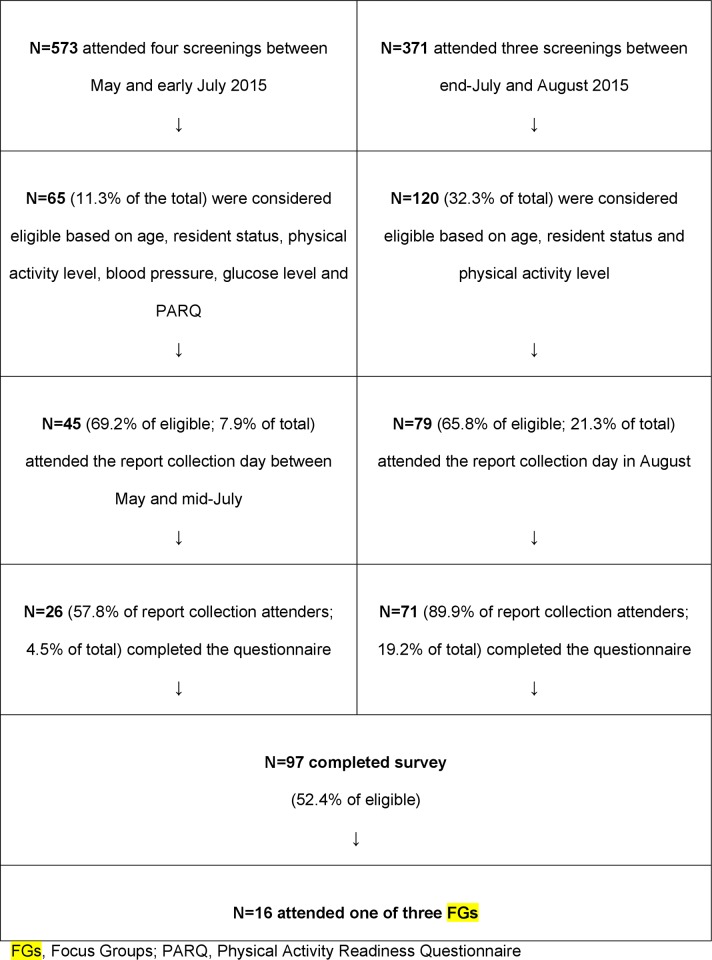
Flow chart of participant recruitment in the Park Prescription Study.

Fig 1 presents the number of people who joined one of the health screening events where we recruited for this study. The figure also reflects eligibility and participation rates of those who were invited to participate in the Park Prescription Study.

Overall, 97 people were willing to participate and all of them completed the questionnaire. Sixteen participants agreed to participate in the FGs. In total, three FGs were conducted, with four to seven participants per group. The average FG duration was 77 minutes. Depending on the participants of each FG, one was conducted in English, one was conducted in Chinese and one was conducted in English and Chinese simultaneously. All FGs were mixed in terms of gender and age, but only one included multiple ethnicities, i.e., Chinese and Indian.

The individual face-to-face interviews with doers were conducted on 3 weekdays and 4 weekend days and each interview took no longer than 15 minutes. No formal record of the number of people approached was made, but between 24 to 27 people were asked to participate. Sixteen of them agreed, reflecting a participation rate of approximately 60%.

Participants’ characteristics for each component are presented in Table 1. The majority of participants were female and of Chinese ethnicity for all components. Eighty-four percent of the participants who completed the questionnaire had an education degree less than or equal to post-secondary education and 23% of them were unemployed.

**Table 1:** Participant characteristics per study component, reflecting total numbers and respective proportions (%), except for age, for which mean±SD or age range are reported.

	Questionnaire (N = 97)	FGs (N = 16)	Doers research (N = 16)
**Mean age (±SD)**^**a**^	54.6 (8.5)	53.2 (8.8)	
**Age range**^**b**^			
40-<55 years			8 (50)
55-<65 years			8 (50)
**Gender N (%)**^**c**^			
Male	37 (38)	7 (44)	8 (50)
Female	60 (62)	9 (56)	8 (50)
**Ethnicity N (%)**^**c**^^,^^**d**^			
Chinese	87 (90)	15 (94)	13 (81)
Malay	5 (5)	0 (0)	2 (13)
Indian	2 (2)	1 (6)	0 (0)
Other	3 (3)	0 (0)	0 (0)
**Marital status N (%)**^**c**^		Not assessed	
Never married	8 (8)		
Currently married	80 (83)		
Separated/widowed/divorced	9 (9)		
**Education N (%)**^**c**^			
No formal/PSLE/less than secondary education	39 (40)		
Secondary/O/N/ITE/NTC	43 (44)		
Post-secondary education/A’level/Poly/Other diploma	12 (12)		
University degree and above	3 (3)		
**Employment status N (%)**^**c**^			
Employed	61 (63)		
Unemployed	22 (23)		
Retired	12 (12)		
Other	2 (2)		

ITE: Institute of Technical Education, NTC: National technical certificate, O/N: O and N levels

Poly: Polytechnic, PSLE: Primary school leaving exam, 12 years old

^a^ For one FG participant, age was not noted

^b^ Participants were purposefully sampled in various age categories

^c^ Percentages may not add up to 100% due to rounding

^d^ For one participant, ethnicity was not reported by the interviewer after the interview.

### Quantitative questionnaire findings

**Fig 2:**
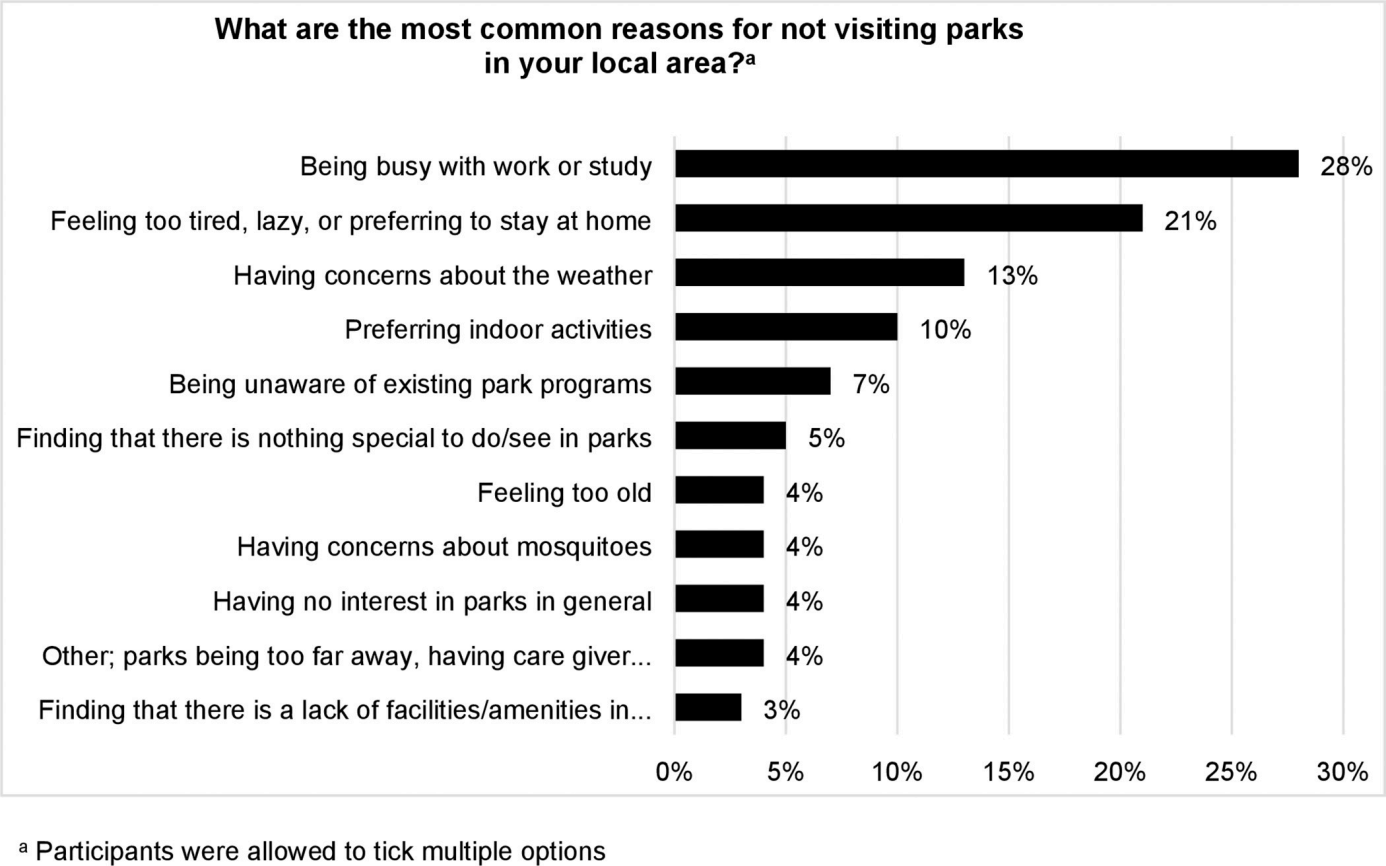
Overview of reasons to not visit parks.

Results from the questionnaire (Table 2) showed that 27 (29%) participants had not visited a park in their local area, while 67 (71%) visited a park at least once in the past month. Fig 2 provides a schematic overview of the participants’ reasons for not visiting parks in their local area. Participants mentioned “being busy with work or study” (28%) most frequently, followed by “feeling too tired, lazy or prefer to stay at home” (21%) and “having concerns about the weather” (13%). Other reasons for not visiting parks expressed by four (4%) participants were distance of parks and having other (priority) duties.

**Table 2:** Current and preferred park use pertaining to physical activities.

	N (%)^a^		N (%)^a^
**Number of times the participants visited a park over the past month (N = 94)**		**Preferred weekly frequency of engaging in park-based physical activities (N = 76)**	
Never	27 (29)	Once	52 (68)
Once or twice	32 (34)	Twice	17 (22)
Three to seven times	23 (24)	More than three times	7 (9)
Eight times or more	12 (13)		
		**Preferred duration of park-based physical activities (N = 88)**	
		About 15–30 minutes per session	59 (67)
		About 45 minutes per session	14 (16)
		60 minutes or more per session	15 (17)
		**Preferred intensity of park-based physical activities (N = 84)**	
		Light intensity only	48 (57)
		Up to moderate intensity	34 (40)
		Up to vigorous intensity	2 (2)
**Type of activities participants engaged in during their last park visit (N = 97)**^**b**^		**Type of activities participants would be interested in to engage in at the park (N = 96)**^**b**^	
Walking with family/friends	42 (43)	Self-guided walking	91 (95)
Walking alone	39 (40)	Walking tour led by guide	68 (71)
Passive activities such as reading, watching children	15 (16)	Yoga	55 (57)
Active sport/activities such as cycling, ball games, martial arts	8 (8)	Aerobic dance	54 (56)
Jogging	7 (7)	Tai-Chi	51 (53)
Walking with dog(s)	2 (2)	Qi-Gong	50 (52)
Gardening	1 (1)	Pilates	45 (47)
		Kickboxing	35 (36)
		Other non-physical activities, such as carnival/fun fair, family bonding, picnic, musical performances, watching people, admiring scenery	12 (13)
		Running/jogging	9 (9)
		Cycling	4 (4)
		Aerobic exercise/routine exercises	3 (3)
		Ball sports	2 (2)
		Line dancing	2 (2)
		Gymnastics	1 (1)
		Wing Chun Kung Fu	1 (1)

^a^ Missing and invalid responses are not reported. For this reason, and/or because of rounding, percentages may not add up to 100%. Reported percentages are calculated based on the total participants who answered the respective item

^b^ Participants were allowed to tick multiple options, hence percentages do not add up to 100%.

The most frequently reported activity during their last park visit was walking, either alone (40%) or with family/friends (43%). Only 15 (15%) participants had engaged in jogging or active sports/activities such as cycling or ball games when they visited the park. One participant stated to use parks for another activity than the ones listed out in the questionnaire, namely gardening.

Fifty-two participants (68%) indicated that they would like to visit the park to engage in physical activities once a week. The majority of participants preferred a duration of park-based physical activities of 15–30 minutes (67%) and with a light intensity only (57%). Participants were interested to engage in a wide variety of park-based physical activities, with the largest proportions of them preferring walking; either self-guided (95%) or led by a guide (71%), Yoga (57%) and/or aerobic dance (56%).

### Qualitative focus groups and doers interview findings

#### Barriers to physical activity

Table 3 provides an overview of all themes and sub-themes as derived from the FGs. In the following sections the numbers of themes and sub-themes correspond to the numbering in this Table overview, but not in chronological order. FG themes that were consistent with quantitative questionnaire findings are highlighted with an asterisk in Table 3.

**Table 3:** Summary of themes and sub-themes derived from the FGs.

Topic	Themes and subthemes
1. Current use of parks	
Park Use	(1i) Using parks for exercise*
	(1ii) Using parks for social interaction*
	**(1iii) Using parks for relaxation and enjoyment**
2. Barriers and facilitators to doing physical activity	
Barriers	**(2i) Intrinsic lack of motivation**
	**(2ii) Comfort-zoning** (also being too tired, too lazy)*
	**(2iii) Risk of injury**
	(2iiia) Traditional Chinese Medicine
	(2iiib) Body ailments
Facilitators	**(2iv) Restorative/invigorating effects of physical activity**
	(2v) Opportunities for social interaction*
	**(2vi) Peer-motivation**
3. Barriers and facilitators to using parks	
Barriers	**(3i) Safety concerns about parks**
	(3ia) Fear of crime
	(3iib) Unsafe park facilities
	(3ii) Haze and hot weather*
	**(3iii) Traveling distance to parks**
Facilitators	**(3iv) Number of facilities**
	**(3v) Scenery and landscape**
4. Preferences for a physical activity program in the park	
Preferred type	(4i) Simple and easy-to-master activities*
	(4ii) Lower intensity activities*
	**(4iii) Family-friendly activities**
	**(4iv) Solitary activities, appealing to males**
Preferred frequency, timing and structure	**(4v) Unstructured events, allowing people to come and go**
	(4va) Multiple sessions a week
	(4vb) Informal learning through ‘community of practice’, or informal group
	**(4vi) Timing at weekday evening and/or weekend morning**

*Consistent with quantitative questionnaire findings

While the importance of physical activity was mostly acknowledged, FG participants mentioned a lack of intrinsic motivation **(2i)** to engage in physical activity if they, for example, did not have a partner: ‘‘*I don’t really exercise due to a lack of exercise partner*. *If I exercise alone*, *it can be quite boring*.*”* (FG1) Some of them also preferred to stay in their comfort zone **(2ii)** rather than to push themselves to go exercise. Participants found it hard to overcome low levels of energy or competing demands such as housework on top of their usual working hours: ‘‘A*fter coming home from work I’m tired and still have to do housework*. *So*, *I’m too tired to exercise*.*”* (FG1) Being lazy was also frequently mentioned in connection with not being active: *‘‘(I exercise) usually on an ‘off-day’ or when I don’t feel lazy*! *Also*, *because there’s no fixed routine or schedule so it’s hard to keep doing it*.” (FG3) The latter quote illustrates that doing physical activity was largely related to occasions when one was free, making it more difficult to sustain between daily routines.

The risk of injuring oneself through physical activity **(2iii)** was a concern with participants who occasionally or regularly engaged in physical activity. For some participants, this risk was shaped by Traditional Chinese Medicine views (**2iiia**), which advocates harmony between the body and the environment. One participant discussed the necessity of a harmonious balance between Yin and Yang forces when doing exercise: *‘‘We should exercise in the morning*, *and only when the sun is out… as the sun would not have risen and there would be too much yin energy in the air*. *So*, *we need the sun to come out first as it would provide yang energy*.” (FG3) Participants who experienced body ailments **(2iiib)** said to cautiously self-monitor and adjust their physical activity intensity accordingly: *‘‘I don’t really intend to increase my physical activity duration or intensity*, *due to my occasional knee pain… if it hurts*, *I just tone down on my activity e*.*g*. *not climb stairs or climb less*.*”* (FG3) They acknowledged the fine balance between being cautious, versus pushing through discomfort for improvement of one’s health. As one participant explained: *‘‘We have to push through*. *With age the ligaments in our legs contract and [we] become less mobile*. *So*, *for me*, *even if I have some pain in the leg I will still force myself to walk… push through the pain–usually I get better*.*”* (FG3)

#### Barriers to park use

During the FGs, three main factors were raised as barriers in frequenting parks. Safety (**3i**) was an important issue in terms of fear of crime (**3ia**) and facilities being perceived as not sufficiently safe **(3ib)**. The presence of shady characters in certain parks made park users feel unsafe at night. Participants described them as potentially dangerous immigrants from Malaysia who stayed at parks overnight: ‘‘*It may be quite scary*. *Because you can imagine if they run out of money they may think about doing something illegal*.” (FG3) Researchers noted how such feelings of danger were contagious, with participants reinforcing each other’s fears in the FGs. Participants with young children also raised concerns about rubber matting at playgrounds, giving off a smell during the hot weather, explaining their worries as follows: ‘‘ […] *under the hot sun in the afternoon I’m not sure if there could be some chemical reaction which poses a safety hazard for young children playing nearby*.” (FG3) Further, it was mentioned that haze (i.e., particulate matter in the air reducing air quality) **(3ii)** could disrupt plans to use the parks, and that as participants valued convenience, long traveling distance to the parks **(3iii)** were deemed unfavorable: ‘*‘Some of the parks are not very convenient*. *You can take a bus there but it takes a long time to walk out […]*. *You need to get to the train*, *sometimes cutting across public housing estates*. *And after that still have to walk to the park so it can be quite frustrating*.” (FG1)

Doers identified different barriers to using parks specifically for exercise. They mentioned park maintenance issues (e.g., falling branches), lighting issues (e.g., parks being too dark), fast riding cyclists and the presence of insects as main factors that made it more difficult for them to exercise in the park. The doers also noted that bad weather occasionally caused their structured outdoor class to be cancelled or resulted in the need to perform physical activity indoors. One interviewee said she felt watched while doing exercises in the parks.

When doers were asked about ways to overcome specific barriers that were identified from the FG participants, they provided the following solutions:

- Set aside time to exercise in the park and commit to your plan. When feeling tired or stressed, see/plan activity in park as time for relaxation;
- Create social support: share your plans with family/friends and get them to remind you. Or involve family/friends in park activities; e.g., spend active time with children/grandchildren;
- In case of bad weather, plan/identify potential alternatives such as going to the gym, climb stairs, walk at void deck areas (open spaces found on the first floor of public housing blocks in Singapore, which may be used for community activities);
- In case of heat, drink plenty of water and seek shade.

#### Current park use, facilitators and benefits

Congruent with the questionnaire findings, walking was considered a popular form of exercise in the context of the park. Participants reported mostly engaging in walking, cycling or playing traditional games, (*e*.*g*., Chapteh, where a weighted shuttlecock is kept in the air usually by the feet) **(1i)**. The restorative effects experienced after exercise **(2iv)** were an important facilitator to engaging in physical activity: *‘‘Personally I feel fresher after exercising; have more energy*. *That’s why I try to exercise at least once a week*.*”* (FG2) Doers shared this perception and mentioned improved health and well-being as the most common benefit of exercising in parks. They also expressed their appreciation for being physically active in the parks specially because they felt close to nature, could breathe in fresh and clean air (as compared to the air-conditioned city environment) and enjoy natural sunlight.

FG participants referred to doing physical activities that offered the opportunity for social interaction **(2v)**, which related to the previously mentioned barrier of an intrinsic lack of motivation to visit parks and exercise. Such social activities provided extrinsic motivation to do physical activity and some participants mentioned the idea of an exercise ‘buddy’ or coach as a form of peer-motivation **(2vi)**. While these participants did not perceive physical activity as important per se, they were willing to take part if pulled along by friends. As explained by one participants: *‘‘I think it’ll be difficult [to encourage me]* … *but if it’s a group walking activity*, *I would consider taking part because my friends are all there”* (FG3).

For other participants, parks were places to people-watch, to learn more about and interact with their community **(1ii)**. FG participants further mentioned to visit parks for relaxation and enjoyment purposes **(1iii)**. Parks with pleasant scenery **(3v)** and sufficient facilities **(3iv)** were perceived as particularly enjoyable. The park aesthetics was reported to be therapeutic for some participants who utilized the space to unwind and engage in introspection.

#### Preferences for a physical activity program in the park

Simple activities that were easy to master appealed specifically to older FG participants who were concerned about difficulties in learning due to their age **(4i)**. Other participants preferred activities of lower intensity **(4ii)**, as they did not want *‘‘To go home with aches and pains*.*”* (FG2) Neuromotor/ balance activities such as Qi Gong, Wing Chun Kung Fu, Tai-Chi or Yoga were popular as they were perceived as relaxing and recreational. Activities with reminiscence value (e.g., childhood games such as ‘Chapteh’), were culturally attractive, with widespread appeal because *‘‘[letting] people re-live their childhood may better motivate them*.” (FG2) A minority of FG participants preferred to sustain their interest through a variety of different classes to pick from each week: *‘‘Because different people have different preferences … offers different things each week for people to try”* (FG2)

Having family-friendly activities **(4iii)** was noted as important for parents, who explained how engaging their child would more likely attract them to these activities: *‘‘We should have activities that are more family or child-oriented so that they can bring their children along and not have to worry what’s going to happen to them*.*”* (FG2) More solitary activities **(4iv)** appealed to male participants who seemed more reluctant to join group-based aerobic activities, perceiving them as mostly for females.

Ideally, participants wanted to be offered multiple sessions a week **(4vi)**, from which to pick and choose. In this way, participants did not have to commit a fixed day to their schedule: *‘‘I mean we already have a fixed schedule for work so I don’t want another rigid thing in my free time*.*”* (FG3) Events taking place on weekday evenings and weekend mornings **(4vb)** and that allowed people to come and go, seemed particularly desirable to the participants **(4v)**.

In addition, participants liked the idea of an informal group **(4vb)** that came together and exercised together. One participant illustrated the ‘carefree’ nature of such an informal approach: ‘‘*All sorts of people who just stop-by to join in the aerobics group; ranging from a well-dressed woman who was on her way to work*, *to an elderly lady just coming back from grocery shopping*. *All of them just put down their stuff*, *took off their sandals or shoes and joined in the activity*.*”* (FG3) Nevertheless, participants acknowledged the difficulties of organizing classes that did not have a fixed schedule and were open to having fixed organized classes all the same. Some participants preferred a fixed venue for familiarity reasons.

## Discussion

This mixed-methods study aimed to inform the development of a Park Prescription intervention, including a face-to-face counseling on physical activity and park use and providing weekly structured exercise sessions in the park to promote physical activity. We managed to recruit a sample of Singaporean adults of low socio-economic status. Participants who completed the questionnaire had low educational levels and all of them lived in public housing (data not shown). A recent review among Singapore citizens showed that staying in public rental housing was an important risk marker for lower participation in health screenings, preference for alternative medicine practitioners and poorer health outcomes [38]. Our results are thus particularly interesting in the context of a hard to reach population most in need of health promotion efforts [39].

Although the majority of the participants indicated that they would like to visit the parks often and engage in physical activity in parks, only few of them went to the park to perform such activities, primarily because they were too busy or too tired. Participants mostly indicated doing informal activities, such as walking, cycling or playing traditional games when using the parks for exercise. They valued parks because of their pleasant landscapes, greenery and facilities and acknowledged the health benefits of parks and natural environments. Participants shared a broad range of barriers to physical activity engagement and park use and provided solutions to overcome certain barriers. Finally, when discussing the content of an exercise-based park program, participants shared their preferences for the type, frequency, duration and timing of activities. The next paragraphs elaborate on the study findings and provide recommendations for the design and content of the Park Prescription intervention.

### Elaboration of findings and implications for the Park Prescription intervention

#### Coping with barriers

The most frequently mentioned barriers to physical activity engagement in general, and to physical activity in parks in specific, were ‘‘being too busy” and ‘‘feeling too tired”. The Centers for Disease Control and Prevention listed the same barriers among the top 10 of most common barriers to physical activity participation [40]. Research among other international populations, including adult Malays, Brazilians and Americans, presented similar results [41–43]. Based on the doers interviews and the FGs, the following strategies could be considered to address these barriers:

- *Emphasize the restorative effect of physical activity and the health benefits of natural environments*. The proposed face-to-face counseling session would offer a good opportunity to discuss barriers with each individual. The counsellor could mention the restorative effect of physical activity and highlight the health benefits of being active in green spaces such as improved well-being and perceiving lower stress [24]. He/she could also share experiences of the individuals’ peers, i.e., those already performing physical activity in parks, as per the peer-to-peer model [44].
- *Encourage goal setting and planning of physical activity*. In their review Greaves and colleagues [45] identified self-regulatory techniques such as goal setting and self-monitoring as possibly effective intervention components associated with changes in physical activity and dietary behaviors. A more recent systematic review and meta-analyses showed that multi-component goal setting interventions (i.e., combining goal setting with other attributes such as strategy planning or feedback) are effective in promoting physical activity across different populations and contexts [46]. Combining goal setting with writing a physical activity plan and keeping track of one’s achievements may thus be a good means to motivate people to incorporate physical activity into their daily lives. This strategy was recommended by our doers interviewees too. Providing a goal setting and/or self-monitoring tool may be considered as part of the Park Prescription intervention.
- *Create social support*. The concept of social support is incorporated in many existing behavior change theories such as the Social Cognitive Theory [47], Social-Ecological Model [48] and the Theory of Planned Behaviour [49], and is believed to positively influence behavior change and maintaining the desired behavior on the longer term. Research has also shown that expanding social networks at an older age is associated with improvements in functional and self-rated health [50]. Finlay and colleagues [51] further found that ageing participants who often live alone, enjoy public green spaces such as parks because they provide opportunities for social interaction and contribute towards their social integration. The Park Prescription intervention will encompass a structured group exercise session in the park, facilitating social interaction among participants in a public green space. The importance of social support from friends or family for realizing behavior change may in addition be a point of discussion during the counseling sessions, as well as the opportunity of enhancing social connections through physical activity in the park.

Singapore has a tropical climate with year-round high and uniform temperatures and high humidity. The country also copes with occasional heavy rainfall and experiences severe periods of haze throughout the year. Research among older adults from six European countries showed that certain weather conditions were associated with physical activity behavior [52]. A recent review and meta-analyses also reported that air pollution discouraged physical activity among adults [53]. The seven studies that were included in the review were either conducted in the US or the United Kingdom. Our results are in line with these findings: weather conditions were an important concern to our participants when considering outdoor physical activities, including exercising in parks. To avoid the heat, the proposed weekly exercise sessions should therefore best be conducted in shady areas and during cooler timings of the day. In addition, it would be beneficial to arrange an indoor space where the sessions can take place in case of rainfall or haze episodes. The latter would also add to the continuity of the sessions. Moreover, any weather-related harmful consequence such as heat stroke, haze hazards or thunder hazards should be clearly communicated to individuals who are participating in the Park Prescription intervention, either during the weekly exercise sessions, the face-to-face counseling or potentially via intervention materials.

The current research showed that the fear of injuring oneself may hinder physical activity engagement in our target group. International studies have documented that fear of injury is a common barrier to physical activity participation, especially among older adults [42,54,55]. To avoid injuries from occurring, and to create a feeling of physical safety among participants of the Park Prescription intervention, a certified doctor and exercise specialist should be consulted during the development of the weekly structured exercise sessions and emphasis be placed on including age-appropriate content. The Park Prescription sessions should ideally be conducted by a trained exercise instructor who has experience working with this age group. He/she should also be made aware that participants have been largely inactive and may not know the limits of their bodies at the start of the intervention. Phillips and colleagues [56] further suggested that education on safety techniques, gradually increasing the duration and frequency of physical activities and focusing on low intensity activities could help to address injury concerns. Such safety principles are similar to those operated within the existing EIM program. Another way of dealing with this specific barrier is to include safety information in the intervention materials for participants and highlight those during the face-to-face counseling.

#### Park proximity and accessibility

Thematic analysis showed that both traveling distance to and the accessibility of parks (by public transport) was a key condition for park use among our participants. This is in line with results from a qualitative review showing that several park attributes among which park proximity is important for encouraging park use [57]. Several quantitative studies, on the other hand, reported mixed findings on the association between park proximity/accessibility and park use and physical activity [58–60]. Although the Park Prescription intervention should try to address park proximity and accessibility based on our qualitative findings, one must bear in mind that it may not be the most effective way to increase the quantitative use of parks for physical activity. With over 400 parks on a surface approximately 700 square kilometers, Singapore is referred to as the ‘City in a Garden’ and offers plenty of opportunities for residents in each part of the country to visit (and be active in) parks. Our participants were not well aware of parks that were close to their home and that could be easily reached. As part of the questionnaire, but not reported on in the current study, we asked participants whether they could name up to 3 parks in their neighborhood. Although two-thirds of the participants wrote down the name of at least one park, there was minor variation among the parks mentioned and several parks appeared not to exist. Participants of the Park Prescription intervention may need to be better informed about parks in their living area in order to use them, including public transport routes and access points. Also, to encourage physical activity in parks specifically, such park information could highlight exercise facilities within the parks. For a good take up of the weekly structured sessions, the parks where the sessions take place should best be organized close to where most of the participants live. Another more general but still relevant factor to consider is the perceived safety of parks (also an important factor for park use according to the qualitative review by McCormack et al. [57]). This may refer to the maintenance of walking paths and exercise equipment, sufficient light at night and/or measures to decrease mosquito breeding, which were worries of our participants in the context of park use. An attempt should be made to take these worries into account when selecting parks for the intervention.

#### Content of the structured-exercise sessions

Participants preferred to engage in low-intensity activities, for about 30 minutes per session. Research on the health benefits of light-intensity physical activity is accumulating [61,62]. Gradually increasing one’s activities, starting at lower intensity levels and slowly increasing to achieve moderate intensity levels may reduce the incidence of adverse events and improve adherence [62]. One activity that would lend itself well in this context is walking. Walking was also the most commonly reported physical activity that participants currently engaged in and would be interested to do. Walking has been shown to be reversely associated with cardio-metabolic risk [63] and mortality [64]. It is regarded as an appropriate activity for any age group. Other activities of interest to our participants included Yoga, Pilates and Qi-gong, Tai-Chi. Benefits for middle-aged and older populations of traditional Asian physical activities such as Yoga are well-documented [65,66] and have been recommended by the Health Promotion Board, Singapore to increase strength and balance in adults, specifically the elderly. These activity types could be taught as part of the weekly exercise sessions.

Participants also expressed their preference for family friendly activities and/or activities with social elements. Research concurrently showed that leisure time activities combining mental, physical and/or social components reduce dementia risk among older adults [67]. It would thus be beneficial to include team challenges or partnered exercises in the Park Prescription exercise sessions. Considering the importance of social support and social interaction for behavior change[47–49] and health [50], participants should further be encouraged to go for walks or exercise in the park with family or friends in their free time. This could be done during the counseling sessions or by highlighting such opportunities in the intervention material.

According to the participants of this study, timing of the weekly exercise sessions is another important condition for their attendance, which is not surprising considering the many work- and family responsibilities most of them experience. Results from the thematic analysis showed that weekday evenings and weekend mornings were suitable timings for most participants. But during the actual FG discussion, it also became evident that catering to each participant’s need would be difficult.

### The Asian context

A key feature of this study is that it took place in an Asian context. Barriers and facilitators of physical activity and park use in our Singapore population were nevertheless fairly similar to those identified by McCormack and colleagues [57], who performed a qualitative review on this topic including research from mainly the US and Australia. Singapore is considered one of the wealthiest and most well-developed nations in the world [68]. This may be a reason why the views and preferences of its inhabitants show resemblance to those of Western populations. Our results did highlight a few focus points that could be considered typical for Singapore:

- The preference to engage in traditional Asian exercises such Yoga, Tai-Chi, Pilates and Qi-Gong was high among our participants and they also mentioned the nostalgia of playing traditional Asian games such as Chapteh in the park. This finding is supported by results form a recent systematic review and meta-analysis which showed that Yoga was among the top-5 leisure time physical activities (ranking fourth after walking, running and cycling) that adults from South East Asia engaged in [69]. Although Asian-based leisure time physical activities such as Yoga are also quite popular among some Western populations, the same review did not list them as common activities among adults from other regions, including Africa, Europe, the Americas, Western Pacific and Eastern Mediterranean [69]. Including traditional Asian activities in park-based physical activity programs may specifically appeal to Singaporeans or Asian populations in general.
- Singapore’s high humidity and heat discouraged our participants from being physically active outdoors. They were often also worried about the air quality. Weather conditions have been associated with outdoor physical activity in older European adults too [52], but unlike our participants, they tended to become more active when temperatures got up. Whether weather conditions hamper or promote physical activity seem to depend on the usual climate and thus on the specific region that adults live in. It may be crucial to the uptake of park-based physical activity among Singaporeans to a) closely monitor the temperature and air quality and inform participants accordingly, and b) provide an indoor option for exercise classes in case of heavy rain or haze.
- A well-maintained park is never far away in Singapore. Park accessibility in this country is very high compared to, for example, estimations from the US where residential populations have to travel an average of about 10 kilometers (6.7 miles) to access their local neighborhood parks [70]. Almost all Singaporeans have a large park in or close by their neighborhood, including exercise corners and walking paths, which offers an excellent opportunity for promoting outdoor park-based exercise in this population.

### Strengths and limitations

To our knowledge, this is the first mixed-methods study looking into the development of a Park Prescription intervention for inactive community members in Asia and beyond. The main strength of the current study is that it combines different research components (questionnaire, FGs and doers interviews) to facilitate an in-depth understanding of important factors such as the personal barriers, enablers and preferences of the target group related to park use and physical activity behavior, which may eventually enhance the effectiveness of the intervention to be developed. We managed to recruit a sample of Singaporean adults with relatively low socio-economic status. Individuals of lower socio-economic status tend to be particularly hard to reach with health promotion efforts, but are most in need of intervention efforts due to their lower physical activity levels [39]. We also acknowledge several study limitations. During some of the report collection events participants engaged in discourse with hospital health screening volunteers about questionnaire items. This might have led to social desirability bias. The questionnaire was adapted from a previously published questionnaire, but not validated in the local context, as no such instrument has been developed and validated with this population. FG participants were recruited from the questionnaire sample, which may have led to social desirability and detection bias. Conducting the FGs in different languages, especially the bilingual one, may have compromised the understanding of some attendees and therefore the quality of data. This procedure was necessary in the multi-ethnic population of Singapore which consists of varied native speakers. The response rate was extremely low despite our best efforts and this reduces the generalizability of our findings. Lastly, this study was conducted among a selective sample of relatively healthy Singapore adults, mostly living in the North of Singapore. Results can therefore not be generalized to less healthy individuals (e.g., those with chronic conditions such as diabetes or heart disease may experience very different barriers, enablers and preferences to physical activity in parks), other age groups, or those living in other parts of Singapore.

## Conclusions

This mixed-methods study showed that community-dwelling individuals in Singapore expressed an intention to visit parks often and be active there, but only few of them do so. The identified important barriers (e.g., being too busy, lack of social support, weather-related concerns and the fear of injuring oneself) and facilitators (e.g., park proximity and accessibility, physical activities of interest to the target group) to physical activity and park use will inform the design of a Park Prescription intervention. The effectiveness of the Park Prescription intervention in promoting physical activity, park use, as well as physical and mental well-being will be tested in a one-year Randomized Controlled Trial.

## Supporting information

S1 Supporting information Questionnaire ENG. (DOCX)

S2 Supporting information Questionnaire CHIN. (DOCX)

S3 Supporting information Questionnaire MAL. (DOCX)

S4 Supporting information FG protocol. (DOCX)

S5 Supporting information Doers interview protocol. (DOCX)
