# Supplementary material for: The Park Prescription Study: Development of a community-based physical activity intervention for a multi-ethnic Asian population
Source: PLoS One. 2019 Jun 11;14(6):e0218247. doi: 10.1371/journal.pone.0218247 (PMC6559668; doi:10.1371/journal.pone.0218247)
Supplement: S1 Supporting information — (DOCX) [file pone.0218247.s001.docx]

**
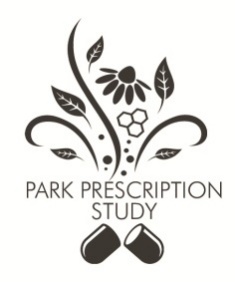
**

**FORMATIVE SURVEY**

|  |
| --- |

***Part A: Sociodemographics***

**1.** Date of birth: ___ ___ / ___ ___ / ___ ___ ___ ___

D D M M Y Y Y Y

**2.** Gender:

🞎 Male

🞎 Female

**3.** Ethnicity:

🞎 Chinese

🞎 Malay

🞎 Indian

🞎 Other, please specify: __________________________________________________

**4.** What is your current marital status?

🞎 Never married

🞎 Currently married

🞎 Separated

🞎 Divorced

🞎 Widowed

**5.** What is the highest level of education you have attained?

🞎 No formal education

🞎 Primary school only

🞎 PSLE

🞎 Secondary school

🞎 ‘O’ or ‘N’ level, or NTC-3 certificate, or equivalent

🞎 ‘A’ level, or NTC-1 or -2 certificate in office or business

🞎 Polytechnic diploma

🞎 Other diploma or professional qualification, please specify: _____________________

🞎 University diploma or above

**6.** Which of the following best describes your main work status in the past 12 months?

🞎 Currently employed, please specify occupation: ______________________________

🞎 Full-time student

🞎 National service

🞎 Retired, please specify previous occupation before retiring: _____________________

🞎 Unemployed, but able to work

🞎 Unemployed, unable to work due to disability or medical condition

**7.** What was the average household earnings (S$) per month in the past 12 months?

🞎 Below S$2,000 per month

🞎 S$2,000-S$3,999 per month

🞎 S$4,000-S$5,999 per month

🞎 S$6,000-S$9,000 per month

🞎 S$10,000 and above per month

🞎 Do not wish to reply

**8.** What type of house do you live in?

🞎 1-/2-room HDB flat

🞎 3-room HDB flat

🞎 4-room HDB flat

🞎 5-room/executive HDB flat

🞎 Private condominium

🞎 Landed house

***Part B: The following questions ask about your physical activity during leisure time. Activities could be for sport, fitness, or other recreational activities. Please answer as honestly and completely as possible – remember this survey is anonymous and there is no “right” or “wrong” answers.***

**9.** Do you do any vigorous-intensity activities for at least 10 minutes continuously?

*Vigorous-intensity aerobic activity causes your heart rate to increase significantly. You are breathing hard and fast and you will find it difficult to hold a conversation with someone (e.g., jogging or running, swimming continuous laps, skipping rope, playing singles tennis).*

🞎 Yes

🞎 No

**10.** In a typical week, on how many days do you do vigorous-intensity activities?

___________days

**11.** How much time do you spend doing vigorous-intensity activities on a typical day?

___________hours _____________ minutes

**12.** Do you do any moderate-intensity activities for at least 10 minutes continuously? *Moderate-intensity aerobic activity causes a slight increase in breathing and heart rate. However, you are still able to talk but not sing during the activity. You should also be perspiring (e.g., brisk walking, leisure cycling, leisure swimming, playing doubles tennis)*

🞎 Yes

🞎 No

**13.** In a typical week, on how many days do you do moderate-intensity activities?

___________days

**14.** How much time do you spend doing moderate-intensity activities on a typical day?

___________hours _____________ minutes

**15.** What is the recommended minimum amount of minutes of physical activity per week that adults should complete for important health benefits?

_____________ minutes

**16.** Do you consider yourself to be currently physically active?

🞎 Yes

🞎 No

**17.** Do you intend to become physically active in the next 6 months?

*If you already consider yourself to be currently physically active, please tick ‘yes’*

🞎 Yes

🞎 No

**18.** Do you currently engage in regular physical activity?

*For activity to be regular, it must add up to a total of 30 minutes or more per day and to be done at least 5 days in a week.*

🞎 Yes

🞎 No

**19.** Have you been regularly physically active for the past 6 months?

🞎 Yes

🞎 No

***Part C: The following questions ask about you and your neighbourhood or local area. Both neighbourhood and local area means everywhere within a 10-15 minute walk from home. Please answer as honestly and completely as possible – remember this survey is anonymous and there is no “right” or “wrong” answers.***

**20a.** Thinking about the past month, how many times did you visit any park in your local area? *Please tick one box only.*

🞎 None 🞎 5 times

🞎 Once 🞎 6 times

🞎 Twice 🞎 7 times

🞎 3 times 🞎 8 times or more

🞎 4 times 🞎 Don’t know

**20b.** What is/are the most common reason(s) for NOT visiting parks in your local area. *Please tick all boxes that apply.*

🞎 Busy with work or study 🞎 Mosquitoes

🞎 Prefer indoor activities 🞎 Weather-related reason

🞎 Too tired, lazy, prefer to stay at home 🞎 Old age

🞎 Nothing special to do/see in the parks 🞎 Unaware of park programs

🞎 Lack of facilities/amenities in the parks 🞎 No interest

🞎 Other, *(specify)* ____________________________________________________

**21.** When you last visited any park in your local area, what did you do during your stay?

*Please tick all boxes that apply.*

🞎 Walking alone

🞎 Walking with family/friends

🞎 Walking with dog(s)

🞎 Jogging

🞎 Active sport (e.g., cricket, football)

🞎 Passive activities (e.g., reading, sitting, watching sport, watching children, picnicking)

🞎 Informal activities (e.g., cycling, ball games, martial arts, meditation)

🞎 Other (*specify)* __________________________________________

**22.** Thinking of parks around your neighbourhood, can you name any parks?

🞎 No

🞎 Yes, please list the places below in rank order that you visit most often, and score each park on their attractiveness and ease of use on a scale of 1-10 (1=poor, 10=best). *Please circle the score that applies.*

Park Name Attractiveness Ease of Use

**1. ________________________ 1-2-3-4-5-6-7-8-9-10 1-2-3-4-5-6-7-8-9-10**

**2. ________________________ 1-2-3-4-5-6-7-8-9-10 1-2-3-4-5-6-7-8-9-10**

**3. ________________________ 1-2-3-4-5-6-7-8-9-10 1-2-3-4-5-6-7-8-9-10**

***Part D: The following questions ask about the PARK YOU VISIT MOST OFTEN – the one you have listed above. When responding to the questions, please keep this park in mind. Please answer as honestly and completely as possible – remember this survey is anonymous and there is no “right” or “wrong” answers.***

**23.** What are the main reason(s) you use this park for your recreational activities?

*Please tick all* *boxes that apply.*

🞎 To get exercise 🞎 To relax, enjoy scenery

🞎 Close to home 🞎 Safety/security

🞎 Large space 🞎 Accessible by car

🞎 Facilities available 🞎 To socialize

🞎 Availability of parking 🞎 Easy to walk to

🞎 Dogs allowed

🞎 Other *(specify)* __________________________________________________

**24.** With whom do you visit this park with?

Relationship to you: _______________________________________________

**25.** What is the ideal travel time for you to reach a park?

**a.** ___________ minutes walking

**b.** ___________ minutes by motorized transport (e.g., bus, MRT, taxi)

***Part E: For the following set of questions, please circle how strongly you agree or disagree with the statements in regards to the PARK YOU VISIT MOST OFTEN. Please only circle one answer. Please answer as honestly and completely as possible – remember this survey is anonymous and there is no “right” or “wrong” answers.***

**26.** I feel safe to walk in this park during day time.

Strongly Somewhat Neutral Somewhat Strongly

Disagree Disagree Agree Agree

**27.** Dogs are welcomed in this park.

Strongly Somewhat Neutral Somewhat Strongly

Disagree Disagree Agree Agree

**28.** This park is attractive to me.

Strongly Somewhat Neutral Somewhat Strongly

Disagree Disagree Agree Agree

**29.** Litter is often present on the park grounds.

Strongly Somewhat Neutral Somewhat Strongly

Disagree Disagree Agree Agree

**30.** The footpaths of this park are well maintained.

Strongly Somewhat Neutral Somewhat Strongly

Disagree Disagree Agree Agree

**31.** Water coolers are conveniently located in this park.

Strongly Somewhat Neutral Somewhat Strongly

Disagree Disagree Agree Agree

**32.** There are shelters and shaded places within the park.

Strongly Somewhat Neutral Somewhat Strongly

Disagree Disagree Agree Agree

**33.** This park is a good place to exercise and do recreational activities.

Strongly Somewhat Neutral Somewhat Strongly

Disagree Disagree Agree Agree

**34.** I enjoy exercising in this park.

Strongly Somewhat Neutral Somewhat Strongly

Disagree Disagree Agree Agree

**35.** This park is a good place to socialize with other people.

Strongly Somewhat Neutral Somewhat Strongly

Disagree Disagree Agree Agree

**36.** I like to spend time with other people in this park.

Strongly Somewhat Neutral Somewhat Strongly

Disagree Disagree Agree Agree

**37.** This park is a good place for community and other social gatherings.

Strongly Somewhat Neutral Somewhat Strongly

Disagree Disagree Agree Agree

***For the following set of question, please circle how interested you are in the activities to be done at PARKS IN GENERAL. Please answer as honestly and completely as possible – remember this survey is anonymous and there is no “right” or “wrong” answers.***

**38.** Self-guided walking tour

Not Interested Somewhat Interested Very

At All Interested Interested

**39.** Walking tour led by tour guide

Not Interested Somewhat Interested Very

At All Interested Interested

**40.** Tai-Chi

Not Interested Somewhat Interested Very

At All Interested Interested

**41.** Qi Gong

Not Interested Somewhat Interested Very

At All Interested Interested

**42.** Kickboxing

Not Interested Somewhat Interested Very

At All Interested Interested

**43.** Yoga

Not Interested Somewhat Interested Very

At All Interested Interested

**44.** Aerobic Dance

Not Interested Somewhat Interested Very

At All Interested Interested

**45.** Pilates
 Not Interested Somewhat Interested Very

At All Interested Interested

**46.** Are there any other activities that you would enjoy doing in parks?

_________________________________________________________________________

_________________________________________________________________________

_________________________________________________________________________

**47.** For the activities mentioned above, how often would you visit parks to engage in those activities?

🞎 None

🞎 Once a week

🞎 Twice a week

🞎 Three times a week or more

**48**. For the activities mentioned above, how long would you do the activities in parks?

🞎 Not at all

🞎 about 15 minutes

🞎 about 30 minutes

🞎 about 45 minutes

🞎 about 60 minutes

🞎 more than 60 minutes

**49**. For the activities mentioned above, at what intensity would you enjoy participating in these activities?

🞎 I would not enjoy participating in any of the activities

🞎 Light intensity only

🞎 Up to moderate intensity

🞎 Up to vigorous intensity
