# Supplementary material for: The Park Prescription Study: Development of a community-based physical activity intervention for a multi-ethnic Asian population
Source: PLoS One. 2019 Jun 11;14(6):e0218247. doi: 10.1371/journal.pone.0218247 (PMC6559668; doi:10.1371/journal.pone.0218247)
Supplement: S2 Supporting information — (DOCX) [file pone.0218247.s002.docx]

**
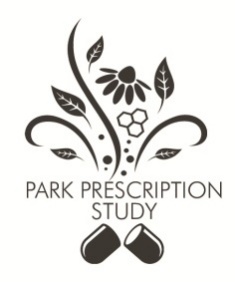
**

**FORMATIVE SURVEY**

|  |
| --- |

***A部分：个人背景资料：***

**1.** 生日: ___ ___ / ___ ___ / ___ ___ ___ ___

日 日 / 月 月 / 年 年 年 年

**2.** 性别

🞎男

🞎女

**3.** 种族：

🞎华族同胞

🞎马来族同胞

🞎印族同胞

🞎其他，请说明: __________________________________________________

**4.** 您目前的婚姻状况是：

🞎未婚

🞎已婚

🞎分居

🞎离婚

🞎寡居

**5.** 您的最高学历是:

🞎没有接受过任何正式教育

🞎接受过小学教育

🞎小六水平

🞎接受过中学教育

🞎拥有‘O’ 或 ‘N’水准，或NTC-3等同文凭

🞎拥有‘A’ 水准　或 NTC-1 或　2 商业文凭

🞎拥有理工学院文凭

🞎拥有其类他文凭或专业资格，请说明: ________________________________

🞎大学文凭或以上

**6.** 在过去12个月里，以下哪个选项最能说明您的工作状态？

🞎受雇人士，请说明现有职业： ______________________________________

🞎全职学生

🞎服役人士

🞎退休人士，请说明退休以前所从事的职业: _____________________________

🞎待业中，但可以正常工作

🞎待业中，因残缺或病状而无法工作。

**7.** 在过去12个月里，您的平均家庭收入是：

🞎每月低于S$2,000

🞎每月介于S$2,000-S$3,999

🞎每月介于S$4,000-S$5,999

🞎每月介于S$6,000-S$9,000

🞎每月S$10,000或以上

🞎不方便回答

**8.** 请问您目前居住在什么样类型的房子里？

🞎一/二房市组屋

🞎三房市组屋

🞎四房市组屋

🞎五房市组屋/执行共管公寓

🞎私人公寓

🞎有地住宅

***B部分：接下来的问题将针对您在平日空闲时间中所进行的体育活动进行调查。这里所指的体育活动包括运动，健身或其他休闲活动。请您提供最诚实并最完整的答案－请记得这项调查是以匿名的方式进行，这些问题也没有任何“对”或“错”的正确解答。***

**9.** 你是否会进行任何至少持续１０分钟以上不间断的剧烈有氧运动？

剧烈有氧运动会使您的心脏跳动率有显著的增加。您将会感到呼吸急促，同时您会发现自己无法正常地与他人保持交谈。（例如：跑步或快跑，游泳，跳绳或单向网球）

🞎 会

🞎 不会

**10.** 在平常的一周里，您有几天会进行剧烈运动？

___________天

**11.** 在您平常进行剧烈运动时，每日会花多少时间做这项运动？

___________小时 _____________分钟

**12.** 您是否会进行任何持续10分钟以上不间断的中等强度运动？

*中等耐力的有氧运动会让您的呼吸与心脏跳动频率略微升高。您依旧能正常说话，但不能在运动时唱歌。同时也应该会因运动而出汗。（例如:快走，慢骑自行车，悠闲地游泳，双人网球）*

🞎 会

🞎 不会

**13.** 在平常的一周里，您有几天会进行中等强度运动？

___________天

**14.** 在平常进行中等强度运动时，您每日会花多少时间做这项运动？

___________小时_____________分钟

**15.** 您认为一般成年人平均每周最少应该进行多久的体能活动才能获取必要的健康效果?

_____________ 分钟

**16.** 您认为自己目前是否拥有充足的运动？

🞎 有

🞎 没有

**17.**在未来的６个月里，您是否打算开始进行充足的运动？

如果您认为您目前已经有足够的运动，请选择“是”。

🞎 是

🞎 不是

**18.**您目前是否定期进行任何体育活动?

定期的体育活动必须是每天至少30分钟或更长时间，一星期至少进行5天。

🞎 有

🞎 没有

**19.**在过去的6个月里，您是否保持进行充足的运动？

🞎 是

🞎 不是

***C部分：接下来的问题将针对您与您所居住的邻里或社区进行调查。您的邻里与社区包括了从您家出发，行驶10到15分钟所能到达的所有地方。请您提供最诚实并最完整的答案－请记得这项调查是以匿名的方式进行，这些问题也没有任何“对”或“错”的标准答案。***

**20a.**回想过去的一个月里，您几次到访过您社区里的任何一个公园？

您只需选择一个选项

🞎没有 🞎 5 次

🞎 1次 🞎 6 次

🞎 2次 🞎 7 次

🞎 3次 🞎 8 次以上

🞎 4次 🞎不知道

**20b.** 如果您对以上的问题 的选择是“没有”，请在以下选项中说明您不到社区公园的理由。请勾选所有符合的答案。

🞎忙于工作或学业 🞎蚊虫

🞎比较喜欢进行室内活动 🞎与天气有关的原因

🞎疲累、懒惰，比较喜欢呆在家里 🞎年纪较大

🞎公园里并没有特别想做或想看的事物 🞎不清楚公园里的各项活动

🞎公园里缺少了该有的设施/设备 🞎没有兴趣

🞎其他 (请说明)_________________________________________

**21.** 请问你上一次到访社区中的任何一个公园时，你在公园内进行过哪些活动？

请勾选所有符合的答案

🞎自己去散步

🞎与家人/好友结伴散步

🞎与宠物狗一同散步

🞎慢跑

🞎体育活动（例如: 板球或足球活动)

🞎静态活动 (例如:阅读、静坐、观赏比赛、看管小朋友玩乐、野餐）

🞎非正式的活动 (例如：骑脚踏车、球类活动、武术、打坐)

🞎其他 (请说明)_________________________________________

**22.** 请您想想您社区周围的公共空间，您是否能写出任何您所熟悉的公园？

🞎 不可以

🞎 可以：请列出您最常到访的公园，并为各公园的吸引力和使用便利程度打分

(1=非常差/ 10=非常好，请在你的答案打个圈)

公园名称 吸引力 使用便利程度

**1. ________________________ 1-2-3-4-5-6-7-8-9-10 1-2-3-4-5-6-7-8-9-10**

**2. ________________________ 1-2-3-4-5-6-7-8-9-10 1-2-3-4-5-6-7-8-9-10**

**3. ________________________ 1-2-3-4-5-6-7-8-9-10 1-2-3-4-5-6-7-8-9-10**

***D部分: 接下来的问题将针对您最常到访的公园 (根据以上您所列出的答案）进行调查。在您回答问题时，请留意您的答案必须针对您在B 部分-第22题所列出的公园而答。请您提供最诚实并最完整的答案－请记得这项调查是以匿名的方式进行，这些问题也没有任何“对”或“错”的标准答案。***

**23.** 以下哪些选项是您选择到这个公园来的主要原因：

请勾选所有的相关答案

🞎做运动 🞎放松，享受风景

🞎离家近 🞎安全／治安良好

🞎宽广的公共空间　 🞎开车就能到达

🞎公园里的设施 🞎参与社交活动

🞎停车方便 🞎步行就能到达

🞎可以携带宠物（例如:小狗）

🞎 其他，请说明：________________________________________

**24.** 您会和谁一起来这个公园？

与您的关系是:___________________________________________

**25.** 您认为到达公园最理想的行驶时间是多久？

**a.** ___________分钟 走路

**b.** ___________分钟机动交通工具（比如：巴士，地铁，計程车）

***E部分: 接下来的这组问题将针对您最常到访的公园进行调查。请根据您的想法，选择最接近您感受的答案。您只需圈起一个答案。请您提供最诚实并最完整的答案－请记得这项调查是以匿名的方式进行，这些问题也没有任何“对”或“错”的标准答案。***

**26.** 我白天在公园内走动时，都感觉到很安全。

强烈反对 不认同 没有意见 认同 强烈认同

**27.** 公园内应该可以携带狗。

强烈反对 不认同 没有意见 认同 强烈认同

**28.** 这个公园对我而言非常有吸引力。

强烈反对 不认同 没有意见 认同 强烈认同

**29.** 公园内的步道上经常堆满垃圾。

强烈反对 不认同 没有意见 认同 强烈认同

**30.** 公园内的步行道都维持的非常好。

强烈反对 不认同 没有意见 认同 强烈认同

**31.** 公园内的饮水机都设在便利的地点。

强烈反对 不认同 没有意见 认同 强烈认同

**32.** 公园内处处都能找到有盖庭院和有盖休息处。

强烈反对 不认同 没有意见 认同 强烈认同

**33.** 这个公园是一个适合运动和进行休闲活动的好地方。

强烈反对 不认同 没有意见 认同 强烈认同

**34.** 我喜欢在公园里做运动。

强烈反对 不认同 没有意见 认同 强烈认同

**35.** 这个公园是一个与他人接触的好地方。

强烈反对 不认同 没有意见 认同 强烈认同

**36.** 我喜欢在这个公园里与他人共享美好时光。

强烈反对 不认同 没有意见 认同 强烈认同

**37.** 这个公园是一个举办社区或社交活动的好地方。

强烈反对 不认同 没有意见 认同 强烈认同

***对于以下这组问题，请注明您对这些活动在公园里进行的好感与兴趣。 请您在最诚实并最完整的答案打个圈－请记得这项调查是以匿名的方式进行，这些问题也没有任何“对”或“错”的标准答案。***

**38.** 自助步行游

没有兴趣 还可以 有兴趣 非常有兴趣

**39.** 由专业向导带领的步行游

没有兴趣 还可以 有兴趣 非常有兴趣

**40.** 太极

没有兴趣 还可以 有兴趣 非常有兴趣

**41.** 气功

没有兴趣 还可以 有兴趣 非常有兴趣

**42.** 搏击操

没有兴趣 还可以 有兴趣 非常有兴趣

**43.** 瑜伽

没有兴趣 还可以 有兴趣 非常有兴趣

**44.** 有氧舞蹈

没有兴趣 还可以 有兴趣 非常有兴趣

**45.** 普拉提
 没有兴趣 还可以 有兴趣 非常有兴趣

**46.** 您还喜欢在公园内进行什么样的活动？

_________________________________________________________________________

_________________________________________________________________________

_________________________________________________________________________

**47.** 为了参与以上所列出的活动，请问您会愿意每星期到访公园几次？

🞎 一次都不愿意

🞎每星期一次

🞎每星期两次

🞎每星期至少三次

**48**. 针对以上所列出的体育活动，你会愿意在公园里花多长时间进行这项活动？

🞎不愿意花时间

🞎约 15分钟

🞎大约30 分钟

🞎大约 45 分钟

🞎大约60 分钟

🞎超过 60分钟

**49**. 针对以上所列出的体育活动，您会喜欢在多大的强度下进行这项活动？

🞎我不会参与任何一项活动。

🞎最高可到达轻微强度

🞎最高可到达中等强度

🞎最高可到达高强度
