# Supplementary material for: The Park Prescription Study: Development of a community-based physical activity intervention for a multi-ethnic Asian population
Source: PLoS One. 2019 Jun 11;14(6):e0218247. doi: 10.1371/journal.pone.0218247 (PMC6559668; doi:10.1371/journal.pone.0218247)
Supplement: S3 Supporting information — (DOCX) [file pone.0218247.s003.docx]

**
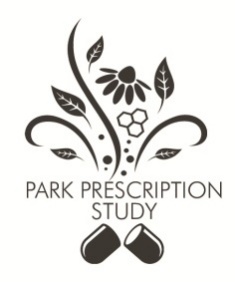
**

**FORMATIVE SURVEY**

|  |
| --- |

***Part A: Sociodemografik***

**1.** Tarikh Lahir : ___ ___ / ___ ___ / ___ ___ ___ ___

H H B B T T T T

**2.** Jantina:

🞎 Lelaki

🞎 Perempuan

**3.** Etnik:

🞎 Cina

🞎 Melayu

🞎 India

🞎 Lain-lain, sila nyatakan: ______________________________________________

**4.** Apakah status perkahwinan anda sekarang?

🞎 Tidak pernah berkahwin

🞎 Masa Ini Berkahwin

🞎 Berpisahan

🞎 Bercerai

🞎 Balu

**5.** Apakah tahap pendidikan tertinggi yang anda telah mencapai?

🞎 Tiada pendidikan formal

🞎 Sekolah rendah sahaja

🞎 PSLE

🞎 Sekolah menengah sahaja

🞎 ‘O’ atau ‘N’ level, atau sijil NTC-3, atau yang setaraf

🞎 ‘A’ level, atau sijil NTC-1 atau -2 dalam pejabat atau perniagaan

🞎 Diploma Politeknik

🞎 Lain diploma atau kelayakan profesional, sila nyatakan : _____________________

🞎 Diploma universiti atau ke atas

**6.** Antara berikut yang manakah paling terbaik menyifatkan status utama kerja anda dalam

tempoh 12 bulan yang lalu?

🞎 Masa ini bekerja, sila nyatakan pekerjaan: ________________________________

🞎 Pelajar sepenuh masa

🞎 Perkhidmatan Negara

🞎 Bersara, sila nyatakan pekerjaan terdahulu sebelum bersara:_________________

🞎 Penganggur, tetapi boleh bekerja

🞎 Penganggur, tidak boleh bekerja kerana kecacatan atau keadaan perubatan

**7.** Apakah pendapatan purata tetangga rumah (S$) setiap bulan dalam tempoh 12 bulan

yang lalu?

 Di bawah S$2,000 sebulan

 S$2,000 - S$3,999 sebulan

 S$4,000 - S$5,999 sebulan

 S$6,000 - S$9,000 sebulan

 S$10,000 dan ke atas setiap bulan

 Tidak ingin menjawab

**8.** Apakah jenis rumah di mana anda tinggal?

 Flat HDB 1- / 2-bilik

 Flat HDB 3-bilik

 Flat HDB 4 bilik

 Flat HDB 5 bilik / eksekutif

 Kondominium Swasta

 Rumah bertanah

***Bahagian B: Soalan-soalan berikut bertanya tentang aktiviti fizikal anda pada masa lapang. Aktiviti boleh untuk sukan, kecergasan, atau aktiviti-aktiviti rekreasi yang lain.*** ***Sila jawab sejujur ​​dan selengkap yang mungkin – Sila ingat kajian ini adalah tanpa nama dan tidak ada jawapan "betul" atau "salah".***

**9.** Adakah anda melakukan apa-apa aktiviti intensiti-lasak sekurang-kurangnya 10 minit secara berterusan? A*ktiviti aerobik intensity-lasak menyebabkan kadar denyutan jantung anda meningkat dengan ketara. Anda bernafas keras dan cepat dan anda akan mendapati sukar untuk mengadakan perbualan dengan seseorang (contohnya, berlari anak atau berlari, berenang pusingan berterusan, tali lompat, bermain tenis perseorangan)*

 Ya

 Tidak

**10.** Dalam masa seminggu, berapa hari yang anda lakukan aktiviti intensiti-lasak?

___________ hari

**11.** Berapa banyak masa yang anda gunakan untuk melakukan aktiviti intensiti-lasak pada

hari biasa?

___________ jam _____________ minit

**12.** Adakah anda melakukan apa-apa aktiviti intensiti-sederhana untuk sekurang-kurangnya 10 minit secara berterusan? A*ktiviti aerobik* intensiti-sederhana *menyebabkan sedikit*

*peningkatan dalam kadar pernafasan dan jantung.* *Walau bagaimanapun, anda masih boleh bercakap tetapi tidak menyanyi semasa aktiviti tersebut.* *Anda juga patut berpeluh (contohnya, berjalan pantas, berbasikal untuk riadah, berenang untuk riadah, bermain tenis beregu)*

 Ya

 Tidak

**13.** Dalam masa seminggu, berapa hari yang anda lakukan aktiviti intensiti-sederhana?

___________ hari

**14.** Berapa banyak masa yang anda gunakan untuk melakukan aktiviti intensiti-sederhana pada hari biasa?

___________ jam _____________ minit

**15.** Berapakah jumlah minit minimum aktiviti fizikal yang disyorkan setiap minggu yang orang dewasa perlu melengkapkan untuk manfaat kesihatan yang penting?

_____________ minit

**16.** Adakah anda menganggap diri anda sebagai aktif secara fizikal pada masa ini?

 Ya

 Tidak

**17.** Adakah anda bercadang untuk menjadi aktif secara fizikal dalam 6 bulan akan datang?

*Jika anda sudah menganggap diri anda aktif secara fizikal pada masa ini, sila tandakan 'ya'*

 Ya

 Tidak

**18.** Adakah anda masa kini melibatkan diri dalam aktiviti fizikal secara kerap?

*Untuk aktiviti menjadi kerap, ia mesti menambah sehingga sejumlah 30 minit atau lebih sehari dan perlu dilakukan sekurang-kurangnya 5 hari dalam seminggu.*

 Ya

 Tidak

**19.** Adakah anda kerap aktif secara fizikal untuk 6 bulan yang lalu?

 Ya

 Tidak

***Bahagian C: Soalan-soalan berikut bertanya tentang anda dan kawasan kejiranan atau setempat anda.*** ***Kedua-dua kawasan kejiranan dan setempat bermakna di mana-mana dalam masa 10-15 minit berjalan kaki dari rumah.*** ***Sila jawab sejujur ​​dan selengkap yang mungkin – sila ingat kajian ini adalah tanpa nama dan tidak ada jawapan "betul" atau "salah".***

**20a.** Berfikir tentang sebulan yang lalu, berapa kali adakah anda melawat mana-mana taman di kawasan tempatan anda? *Sila tandakan satu kotak sahaja.*

🞎 Tiada 🞎 5 kali

🞎 Satu kali 🞎 6 kali

🞎 Dua kali 🞎 7 kali

🞎 3 kali 🞎 8 kali atau lebih

🞎 4 kali 🞎 Tidak tahu

**20b.** Sila tandakan alasan-alasan anda untuk TIDAK melawat mana-mana taman di kawasan tempatan anda. *Sila tandakan semua kotak yang berkenaan.*

 Sibuk dengan kerja atau kajian   Nyamuk

 Lebih suka aktiviti dalaman   Sebab Berkaitan Cuaca

 Terlalu letih, malas, lebih suka tinggal di rumah  Umur Tua

 Tiada apa istimewa untuk dibuat/lihat di taman   Tidak mengetahui program taman

 Kekurangan kemudahan / kemudahan di taman  Tiada minat

 Lain-lain *(nyatakan)* __________________________________________

**21.** Apabila anda dahulu melawat mana-mana taman di kawasan tempatan anda, apa yang anda lakukan sepanjang penginapan anda? *Sila tandakan semua kotak yang berkenaan.*

 Berjalan sahaja

 Berjalan bersama keluarga / rakan-rakan

 Berjalan dengan anjing

 Berlari-lari anak

 Sukan aktif (contohnya, kriket, bola sepak)

 Aktiviti pasif (contohnya, membaca, duduk, menonton sukan, menonton anak-anak,

berkelah)

 Aktiviti tidak formal (contohnya, berbasikal, permainan bola, seni mempertahankan

diri, meditasi)

 Lain-lain *(nyatakan)* __________________________________________

**22.** Memikirkan taman di sekitar kawasan kejiranan anda, bolehkah anda namakan mana-

mana taman ?

 Tidak

 Ya, sila senaraikan tempat-tempat di bawah mengikut pangkat yang anda lawati paling kerap, dan skor setiap taman untuk daya tarikan dan kemudahan penggunaan mereka pada skala 1-10 (1 = tidak baik, 10 = terbaik) Sila bulatkan jawapan anda.

Nama Taman Daya Tarikan Kemudahan Penggunaan

**1. ________________________ 1-2-3-4-5-6-7-8-9-10 1-2-3-4-5-6-7-8-9-10**

**2. ________________________ 1-2-3-4-5-6-7-8-9-10 1-2-3-4-5-6-7-8-9-10**

**3. ________________________ 1-2-3-4-5-6-7-8-9-10 1-2-3-4-5-6-7-8-9-10**

***Bahagian D: Soalan-soalan berikut bertanya tentang TAMAN YANG ANDA LAWAT DENGAN KERAP SEKALI - yang anda telah senaraikan di atas.*** ***Ketika menjawab soalan-soalan, sila simpan taman ini dalam fikiran.*** ***Sila jawab sejujur ​​dan selengkap mungkin – sila ingat kajian ini adalah tanpa nama dan tidak ada jawapan "betul" atau "salah".***

**23.** Apakah sebab utama anda menggunakan taman ini untuk aktiviti rekreasi anda?

*Sila tandakan semua* *kotak yang berkenaan.*

 Untuk mendapatkan latihan   Untuk berehat, nikmati pemandangan

 Dekat dengan rumah   Keselamatan / sekuriti

 Ruang besar   Boleh diakses melalui kereta

 Kemudahan yang disediakan   Untuk bersosial

 Ketersediaan tempat letak kereta   Mudah dicapai dengan berjalan kaki

 Anjing dibenarkan

 Lain-lain *(nyatakan)* __________________________________________________

**24.** Dengan siapakah anda melawat taman ini?

Hubungan dengan anda:

______________________________________________________

**25.** Apakah masa perjalanan yang terbaik bagi anda untuk mencapai sebuah taman?

**a.** ___________ minit dengan berjalan kaki

**b.** ___________ minit dengan kendaraan (bas, MRT, teksi)

***Bahagian E: Untuk set soalan berikut, sila bulatkan berapa kuatnya anda bersetuju atau tidak bersetuju dengan kenyataan berkenaan TAMAN YANG ANDA LAWAT DENGAN KERAP SEKALI.*** ***Sila bulatkan hanya satu jawapan.*** ***Sila jawab sejujur ​​dan selengkap mungkin – sila ingat kajian ini adalah tanpa nama dan tidak ada jawapan "betul" atau "salah".***

**26.** Saya berasa selamat untuk berjalan di taman ini pada waktu siang.

Sangat Agak Neutral Agak Sangat

Tidak Setuju Tidak Setuju Setuju Setuju

**27.** Anjing adalah dialu-alukan di taman ini.

Sangat Agak Neutral Agak Sangat

Tidak Setuju Tidak Setuju Setuju Setuju

**28.** Taman ini adalah menarik kepada saya.

Sangat Agak Neutral Agak Sangat

Tidak Setuju Tidak Setuju Setuju Setuju

**29.** Sampah sering terdapat di kawasan taman.

Sangat Agak Neutral Agak Sangat

Tidak Setuju Tidak Setuju Setuju Setuju

**30.** Laluan pejalan kaki di taman ini dijaga dengan baik.

Sangat Agak Neutral Agak Sangat

Tidak Setuju Tidak Setuju Setuju Setuju

**31.** Penyejuk air turut terletak dengan mudah di taman ini.

Sangat Agak Neutral Agak Sangat

Tidak Setuju Tidak Setuju Setuju Setuju

**32.** Terdapat tempat perlindungan dan tempat-tempat teduh di dalam taman.

Sangat Agak Neutral Agak Sangat

Tidak Setuju Tidak Setuju Setuju Setuju

**33.** Taman ini adalah tempat yang baik untuk menjalankan dan melakukan aktiviti rekreasi.

Sangat Agak Neutral Agak Sangat

Tidak Setuju Tidak Setuju Setuju Setuju

**34.** Saya suka bersenam di taman ini.

Sangat Agak Neutral Agak Sangat

Tidak Setuju Tidak Setuju Setuju Setuju

**35.** Taman ini adalah tempat yang baik untuk bersosial dengan orang lain.

Sangat Agak Neutral Agak Sangat

Tidak Setuju Tidak Setuju Setuju Setuju

**36.** Saya suka menghabiskan masa dengan orang lain di taman ini.

Sangat Agak Neutral Agak Sangat

Tidak Setuju Tidak Setuju Setuju Setuju

**37.** Taman ini adalah tempat yang baik untuk komuniti dan perhimpunan sosial yang lain.

Sangat Agak Neutral Agak Sangat

Tidak Setuju Tidak Setuju Setuju Setuju

***Untuk set soalan berikut ini, sila bulatkan betapa minatnya anda dalam aktiviti-aktiviti yang perlu dilakukan di TAMAN-TAMAN SECARA UMUM.*** ***Sila bulatkan hanya satu jawapan.*** ***Sila jawab sejujur ​​dan selengkap mungkin – sila ingat kajian ini adalah tanpa nama dan tidak ada jawapan "betul" atau "salah".***

**38.** Lawatan berjalan kaki berpandu sendiri

Tidak Berminat Agak Berminat Sangat

Sama Sekali Berminat Berminat

**39.** Lawatan berjalan kaki yang diketuai oleh pemandu pelancung

Tidak Berminat Agak Berminat Sangat

Sama Sekali Berminat Berminat

**40.** Tai-Chi

Tidak Berminat Agak Berminat Sangat

Sama Sekali Berminat Berminat

**41.** Qi Gong

Tidak Berminat Agak Berminat Sangat

Sama Sekali Berminat Berminat

**42.** Kickboxing

Tidak Berminat Agak Berminat Sangat

Sama Sekali Berminat Berminat

**43.** Yoga

Tidak Berminat Agak Berminat Sangat

Sama Sekali Berminat Berminat

**44.** Tarian aerobik

Tidak Berminat Agak Berminat Sangat

Sama Sekali Berminat Berminat

**45.** Pilates

Tidak Berminat Agak Berminat Sangat

Sama Sekali Berminat Berminat

**46.** ​​Adakah terdapat apa-apa aktiviti lain yang anda akan menikmati melakukan di taman-taman?

_________________________________________________________________________

_________________________________________________________________________

_________________________________________________________________________

**47.** Bagi aktiviti yang disebut di atas, berapa kerap anda akan melawat taman-taman untuk melibatkan diri dalam aktiviti-aktiviti tersebut?

 Tiada

 Sekali seminggu

 Dua kali seminggu

 Tiga kali seminggu atau lebih

**48.** Untuk aktiviti yang disebut di atas, berapa lama anda akan melakukan aktiviti di taman-taman?

 Tidak sama sekali

 kira-kira 15 minit

 kira-kira 30 minit

 kira-kira 45 minit

 kira-kira 60 minit

 lebih daripada 60 minit

**49.** Untuk aktiviti yang disebut di atas, pada intensiti apakah yang anda akan menikmati mengambil bahagian dalam aktiviti-aktiviti ini?

 Saya tidak akan menikmati mengambil bahagian dalam mana-mana aktiviti

 Intensiti ringan sahaja

 Sehingga intensiti sederhana

 Sehingga intensiti bertenaga
