# Supplementary material for: The Park Prescription Study: Development of a community-based physical activity intervention for a multi-ethnic Asian population
Source: PLoS One. 2019 Jun 11;14(6):e0218247. doi: 10.1371/journal.pone.0218247 (PMC6559668; doi:10.1371/journal.pone.0218247)
Supplement: S4 Supporting information — (DOCX) [file pone.0218247.s004.docx]

**Procedure:**

1. Greet participants

2. Obtain verbal consent from each of the participants before proceeding with the focus group discussion.

- All participants need to be given the participant information sheet and ample time to read and ask questions prior to the discussion.
- Verbal consent can be taken while seated. All participants need to state their pseudonyms and that they give “consent to participate in this discussion”.

3. Collect anonymous sociodemographic data

- Once verbal consent has been obtained, a research will ask the participant confidentially state their age, gender, and ethnicity. The researcher will then make note of this on a sheet. No names or identifying information will be recorded.

4. Moderator’s Opening

[Begin audio recording]

5. Moderated Discussion

6. Moderator’s Closing

[Stop audio recording]

7. Debriefing between moderator and note-taker

**Moderator’s Opening:**

Introduction [read to participants]:

*Hello, everyone. Thank you for coming today and participating in this focus group discussion. We are interested in understanding physical activity and parks and how parks could be better used for physical activity. For physical activity, we mean any activity that gets your body moving and requires energy, such as exercising. As this is an open discussion, you are each entitled to your opinions, and there is no right or wrong response. We want to hear everyone’s thoughts, and it would be best if you each spoke clearly, one at a time. We will try not to disrupt the discussion, but we may have to shift topics to make sure all topics will be covered. Today’s discussion will be audio recorded, and in order for each person to be identified, we ask that you pick a pseudonym – you do not have to use your real name. This discussion is completely voluntary and you can withdraw from the discussion at any time. If there are questions you do not want to answer, you may choose not to do so. I’d also like to remind you that all the information discussed today will be kept confidential and only the research team will have access to the recording. You may pick to use another name, nickname, such as a pseudonym, or you can make up a whole new name for yourself. I will only address you by your pseudonym so that your participation is anonymous and confidential. Please remember that all information discussed here today will only be used for research purposes. Therefore, it is important to us that you provide your honest thoughts and opinions as it will greatly help us.*

*Do you have any questions about the process before we proceed? I can answer them now before we start the recording. Once we start the recording, I will ask each of you to identify yourself by your pseudonym and verbally consent to the discussion.*

**[Start recording]**

*My name is* ***[Moderator’s Name]*** *and I will be facilitating this focus group discussion today* ***[Today’s Date].***

Go around the table and ask participants to:

1. Identify themselves using pseudonyms only

2. State into the recorder “I consent to this focus group discussion”

**Moderated Discussion:**

Note: Questions and discussions may not appear in this order.

Topic: Physical Activity (PA)

- Domain 1: Perceived current PA and health
  - Q1: Do you consider yourself physically active? In other words, do you exercise?
    - Q1a: if yes, what physical activities or exercise do you currently do?
    - Q1b: If no, what are some possible reasons for not being physically active or exercising?
  - Q1: Are there any potential health benefits of PA or exercise?
    - Q1a: Are you aware of any recommendations by health professionals about the health benefits of physical activity? Such as the type of PA or duration of PA.
- Domain 2: Intent to increase PA
  - Q1: How would you define “regular PA/exercise”?
  - Q2: Based on our discussion of the previous topics (current status of PA and potential health benefits of PA), are you interested in increasing your PA/exercise or engage in “regular PA/exercise”?
    - Q2a: What are some strategies or ways that would help you achieve this?
    - Q2b: What would motivate you to increase your PA/exercise?
  - Q2: What types of PA interests you? These could be completely new activities for you, or activities you have already tried; they could also be done in a group or on your own.
- Domain 3: Barriers to increasing PA
  - Q1a: Are there any reasons that prevent you from engaging in activity?
  - Q1b: Do you have any potential health concerns limiting you to engage in PA?

*Thank you for taking the time to discuss physical activity. Now I would like to switch topics to focus on activity specific in parks.*

Topic: Parks for PA

- Domain 1: Neighborhood parks
  - Q1: Are there parks in your area that could be used for physical activity?
    - Q1a: Is it convenient for you to get to?
  - Q2: Would you consider going to these parks for physical activity? Either by yourself or with a friend.
- Domain 2: PA programs within parks
  - Q1: What are some types of physical activities within the parks that would interest you?
    - Q1a: Should these activities or programs be structured or unstructured?
      - Structured programs, such as instructor led classes
      - Unstructured activities that you can do on your own, such as walking, cycling, using fitness equipment
    - Q1b: How long would you be interested in attending this activity each time?
      - 30 minutes, 1 hour, more than 1 hour.
    - Q1c: How many times a week would you attend this activity?
    - Q1d: Would you prefer to do these activities on weekdays or weekends?
      - If weekend – which part of the day (morning/evening/either) would you prefer; state your preferred starting time (e.g. 7am, 8am, 5pm, 6pm, etc)
      - If weekday – which part of the day (morning /evening/either ) would you prefer; state your preferred starting time; state your preferred starting time (e.g. 7am, 8am, 5pm, 6pm, etc)
    - Q1e: What kind of intensity should these activities be?
      - Should the intensity increase as the weeks go by for the program?
      - *Moderate-intensity aerobic activity causes a slight increase in breathing and heart rate. However, you are still able to talk but not sing during the activity. You should also be perspiring (e.g., brisk walking, leisure cycling, leisure swimming, playing doubles tennis)*
      - *Vigorous-intensity aerobic activity causes your heart rate to increase significantly. You are breathing hard and fast and you will find it difficult to hold a conversation with someone (e.g., jogging or running, swimming continuous laps, skipping rope, playing singles tennis)*
    - Q1f: Should the frequency and duration of these activities increase with the program?
  - Q2: We have a sample here of a “park prescription” that we will pass around for you to review. We would like for you to make some comments and suggestions on this as this will be given to our participants.
    - Q2a: What do you think of the size of the page?
    - Q2b: What do you think of the content included on the “prescription”?
    - Q2c: What do you think of the visual aesthetics?
    - Q2d: Do you have any suggestions to improve the appeal of the prescription, Either aesthetics or content?
- Domain 3: Barriers of PA within parks
  - Q1: Do you have any concerns or worries about doing physical activities in the parks?
    - Q1a: What are some ways to help alleviate these concerns? Either from your perspective and things you can do, or from our (NParks) perspective and what we can provide within the parks

**Moderator’s Closing:**

*Thank you very much for sharing your views on these topics. Your participation is greatly appreciated. The responses you have provided will be very useful to our research in understanding physical activity, parks, and how to best increase physical activity within the parks.*

**[Stop Recording]**
