# Supplementary material for: The Park Prescription Study: Development of a community-based physical activity intervention for a multi-ethnic Asian population
Source: PLoS One. 2019 Jun 11;14(6):e0218247. doi: 10.1371/journal.pone.0218247 (PMC6559668; doi:10.1371/journal.pone.0218247)
Supplement: S5 Supporting information — (DOCX) [file pone.0218247.s005.docx]

**Introduction**

‘It is so nice to see you exercising/being active in the park’.

We try to promote exercise/physical activity in the park in people your age.

Therefore, we are conducting a research study.

Do you mind if I explain you about the study and ask you whether you are interested to participate? It will only take 15 minutes of your time.

*Note:*

- *The interviewer should be able to identify him/herself as being from the university.*
- *The interviewer should hand over the Information Letter to the participant and briefly explain the study to him/her.*
- *Verbal consent should be taken before asking the items.*
- *No audio recordings should be made.*

*The interviewer can take notes during the interview, or just discuss the items with the participants and memorize the key points. The key points should be written down afterwards.*

- *Participant’s age, gender and ethnicity have to be noted.*

**Matrix**

The interviewer should use the two matrices below to select the participants.

All cells should be crossed-off.

1. Recruitment from Sunday’s at the Park; structured-exercise.

If possible in one of the parks we target for the Park Prescription Trial

| Male | Male | Female | Female |
| --- | --- | --- | --- |
| 40<55 | ≥55-65 | 40<55 | ≥55-65 |
| Male | Male | Female | Female |
| 40<55 | ≥55-65 | 40<55 | ≥55-65 |

Total = 8 short interviews

Duration = Expect to take up 15 min per participant

Sampling time = Sunday morning, after Sunday’s at the Parks

2. Recruitment from one of the parks we target for the Park Prescription Trial; free physical activity. Includes walking (preferably at a brisk pace), running, bicycling, playing active games etc.

| Male | Male | Female | Female |
| --- | --- | --- | --- |
| 40<55 | ≥55-65 | 40<55 | ≥55-65 |
| Male | Male | Female | Female |
| 40<55 | ≥55-65 | 40<55 | ≥55-65 |

Total = 8 short interviews

Duration = Expect to take up 15 min per participant

Sampling time = Weekday mornings/afternoons

**Items**

*Benefits/reinforcing factors*

1. What do you see as the benefits, advantages or good things that happen from exercising/being active in the park?
2. Can you tell me about how you compared these to the disadvantages?

*Barriers*

1. What makes it (more) difficult for you to exercise/be active in the park?

*Enabling factors/self-efficacy*

1. What makes it easier for you to exercise/be active in the park?

*Derived from focus groups*

1. Can you tell me how you manage to overcome:
   1. Lack of time to exercise/be active in the park
   2. Feeling too tired to exercise/be active in the park
   3. Concerns with regard to the weather (hot/haze)

**Wrap up**

Finish with thanking the participant and hand over the token.
